# Supplementary material for: Metagenomic sequencing of post-mortem tissue samples for the identification of pathogens associated with neonatal deaths
Source: Nat Commun. 2023 Sep 4;14:5373. doi: 10.1038/s41467-023-40958-8 (PMC10477270; doi:10.1038/s41467-023-40958-8)
Supplement: Supplementary file 1 — Supplementary Information [file 41467_2023_40958_MOESM1_ESM.pdf]

Supplementary Table 1: Characteristics of controls in whom an infectious illness was not attributed in the causal pathway to death.

Control number	**Age in days**	**Antemortem antibiotic use**	**Underlying CoD***	**Immediate CoD***
1	2	None	Birth asphyxia and trauma	Hypoxic ischemic encephalopathy
2	1	None	Prematurity	RDS of Newborn
3	4	None	Prematurity	RDS of Newborn
4	1	None	Prematurity	RDS of Newborn
5	1	None	Birth asphyxia and trauma	Meconium aspiration
6	2	Ampicillin and Gentamycin	Prematurity	RDS of Newborn
7	3	Ampicillin and Gentamycin	Congenital anomalies	Birth asphyxia and trauma
8	<1	Ampicillin and Gentamycin	Prematurity	RDS of Newborn
9	2	Ampicillin and Gentamycin	Congenital anomalies	Anoxic Brain damage
10	4	None	Prematurity	RDS of Newborn
11	2	None	Prematurity	Birth asphyxia and trauma
12	1	Ampicillin and Gentamycin	Birth asphyxia and trauma	Severe Hypoxic ischemic encephalopathy
13	<1	Ampicillin and Gentamycin	Birth asphyxia and trauma	Hypoxic ischemic encephalopathy
14	4	None	Birth asphyxia and trauma	Hypoxic ischemic encephalopathy
15	<1	Ampicillin and Gentamycin	Prematurity	RDS of Newborn
16	1	Ampicillin and Gentamycin	Prematurity	RDS of Newborn

*As determined by the Determination of Cause of Death (DeCoDe) panel of experts who reviewed all available medical and laboratory reports (both premortem and postmortem). All antemortem and postmortem culture and NAAT were negative except for Control number 2, 6 and 16 which cultured Coagulase negative staphylococcus which is a common skin colonizer and were considered by the DeCoDe panel to be contaminates unless detected in multiple cultures pre and postmortem and/or had histological evidence of infection. Abbreviations: RDS – respiratory distress syndrome

Supplementary Table 2: The antemortem and postmortem laboratory findings of neonatal deaths attributed to infections by an expert panel

Case number	Month of Death	Age in days	Clinician diagnosed CoD	Antibiotic use antemortem	Antemortem sample			Post-mortem samples							
					Blood culture			Lung culture			Blood culture			NAAT	
					Isolate	Antimicrobial susceptible	Antimicrobial resistance profile	Isolate	Antimicrobial susceptible	Antimicrobial resistance profile	Isolate	Antimicrobial susceptible	Antimicrobial resistance profile	Lung NAAT	Blood NAAT
1	Aug-16	25-30	Nosocomial sepsis, unspecified bacteria	Ampicillin<br>Gentamycin<br>Tazobactam<br>Amikacin<br>Meropenem<br>Vancomycin	Negative			*A.baum*	Colistin	Amikacin<br>Cefepime<br>Ceftazidime<br>Ciprofloxacin<br>Gentamycin<br>Impenem<br>Meropenem<br>Piperacillin-Tazobactam<br>Tobramycin<br>Trimethoprim-sulfamethoxazole	*A.baum*	Colistin	Amikacin<br>Cefepime<br>Ceftazidime<br>Ciprofloxacin<br>Gentamycin<br>Impenem<br>Meropenem<br>Piperacillin-Tazobactam<br>Tobramycin<br>Trimethoprim-sulfamethoxazole	Negative	Negative
2	Aug-16	15-20	Nosocomial bacterial meningitis	Tazobactam<br>Amikacin<br>Ciprobay<br>Meropenem	*K.pneu*	Meropenem	Ampicillin/Amoxicillin<br>Amoxicillin-clavulanic acid<br>Cefotaxime/Ceftriaxone<br>Ceftazidime<br>Cefepime<br>Gentamicin<br>Tobramycin	*K.pneu*	Amikacin<br>Cefoxitin<br>Ertapenem<br>Imipenem<br>Meropenem	Ampicillin/Amoxicillin<br>Amoxicillin-clavulanic acid<br>Ciprofloxacin<br>Cefazolin<br>Cefuroxime (Parenteral)<br>Cefotaxime/Ceftriaxone<br>Ceftazidime<br>Cefepime<br>Chloramphenicol<br>Gentamicin<br>Tobramycin<br>Trimethoprim-sulfamethoxazole	*A.baum*	Amikacin<br>Ceftazidime<br>Cefepime<br>Colistin	Ciprofloxacin<br>Gentamycin<br>Impenem<br>Meropenem<br>Piperacillin-Tazobactam<br>Tobramycin<br>Trimethoprim-sulfamethoxazole	*K.pneu*	Negative
3	Oct-16	5-10	Sepsis of newborn, gram negative bacteria	Ampicillin<br>Gentamycin<br>Tazobactam<br>Amikacin<br>Vancomycin	*A.baum*	Colistin	Amikacin<br>Cefepime<br>Ceftazidime<br>Ciprofloxacin<br>Gentamycin<br>Impenem<br>Meropenem<br>Piperacillin-Tazobactam<br>Tobramycin<br>Trimethoprim-sulfamethoxazole	*A.baum*	Colistin	Amikacin<br>Cefepime<br>Ceftazidime<br>Ciprofloxacin<br>Gentamycin<br>Impenem<br>Meropenem<br>Piperacillin-Tazobactam<br>Tobramycin<br>Trimethoprim-sulfamethoxazole	*A.baum*	Colistin	Amikacin<br>Cefepime<br>Ceftazidime<br>Ciprofloxacin<br>Gentamycin<br>Impenem<br>Meropenem<br>Piperacillin-Tazobactam<br>Tobramycin<br>Trimethoprim-sulfamethoxazole	*E.coli, RSV*	Negative
4	Oct-16	5-10	Gram negative nosocomial sepsis	Ampicillin<br>Gentamycin<br>Tazobactam<br>Amikacin<br>Vancomycin	*A.baum*	Colistin	Amikacin<br>Cefepime<br>Ceftazidime<br>Ciprofloxacin<br>Gentamycin<br>Impenem<br>Meropenem<br>Piperacillin-Tazobactam<br>Tobramycin<br>Trimethoprim-sulfamethoxazole	*A.baum*	Colistin	Amikacin<br>Cefepime<br>Ceftazidime<br>Ciprofloxacin<br>Gentamycin<br>Impenem<br>Meropenem<br>Piperacillin-Tazobactam<br>Tobramycin<br>Trimethoprim-sulfamethoxazole	*A.baum*	Colistin	Amikacin<br>Cefepime<br>Ceftazidime<br>Ciprofloxacin<br>Gentamycin<br>Impenem<br>Meropenem<br>Piperacillin-Tazobactam<br>Tobramycin<br>Trimethoprim-sulfamethoxazole	*Ureaplasma*	Negative
5	Nov-16	15-20	Nosocomial lobar pneumonia, unspecified bacteria	Ampicillin<br>Gentamycin<br>Tazobactam<br>Amikacin<br>Ertapenem<br>Vancomycin	*A.baum*	Colistin	Amikacin<br>Cefepime<br>Ceftazidime<br>Ciprofloxacin<br>Gentamycin<br>Impenem<br>Meropenem<br>Piperacillin-Tazobactam<br>Tobramycin<br>Trimethoprim-sulfamethoxazole	*A.baum*	Colistin	Amikacin<br>Cefepime<br>Ceftazidime<br>Ciprofloxacin<br>Gentamycin<br>Impenem<br>Meropenem<br>Piperacillin-Tazobactam<br>Tobramycin<br>Trimethoprim-sulfamethoxazole	*A.baum*	Colistin	Amikacin<br>Cefepime<br>Ceftazidime<br>Ciprofloxacin<br>Gentamycin<br>Impenem<br>Meropenem<br>Piperacillin-Tazobactam<br>Tobramycin<br>Trimethoprim-sulfamethoxazole	Negative	Negative
6	Jan-17	15-20	Gram negative nosocomial sepsis	Ampicillin<br>Gentamycin<br>Tazobactam<br>Amikacin<br>Vancomycin	*A.baum*	Colistin	Amikacin<br>Cefepime<br>Ceftazidime<br>Ciprofloxacin<br>Gentamycin	*A.baum*	Colistin	Amikacin<br>Cefepime<br>Ceftazidime<br>Ciprofloxacin<br>Gentamycin	*A.baum*	Colistin	Amikacin<br>Cefepime<br>Ceftazidime<br>Ciprofloxacin<br>Gentamycin	Negative	*S.aur, ureaplasma*

							Impenem<br>Meropenem<br>Piperacillin-Tazobactam<br>Tobramycin<br>Trimethoprim-sulfamethoxazole			Impenem<br>Meropenem<br>Piperacillin-Tazobactam<br>Tobramycin<br>Trimethoprim-sulfamethoxazole			Impenem<br>Meropenem<br>Piperacillin-Tazobactam<br>Tobramycin<br>Trimethoprim-sulfamethoxazole		
7	Jan-16	<1	Sepsis of newborn due to E.coli	Ampicillin<br>Gentamycin	E.coli	Cefotaxime/<br>Ceftriaxone		Negative			*E.coli*	Amikacin<br>Cefazolin<br>Cefepime<br>Cefotaxime/<br>Ceftriaxone<br>Cefoxitin<br>Chloramphenicol<br>Ertapenem<br>Gentamycin<br>Imipenem<br>Meropenem	Amoxicillin-clavulanic acid<br>Ampicillin<br>Ciprofloxacin<br>Nalidixic acid	*E.coli*	*E.coli*
8	Feb-16	1-5	Sepsis of newborn due to S.aureus and other gram negative bacteria	Ampicillin<br>Gentamycin<br>Tazobactam<br>Amikacin	*S.aur*	Clindamycin<br>Fusidic acid<br>Linezolid<br>Rifampicin<br>Vancomycin	Chloramphenicol<br>Ciprofloxacin<br>Cloxacillin<br>Erythromycin/ Azithromycin<br>Gentamycin<br>Penicillin/Ampicillin<br>Trimethoprim-sulfamethoxazole	*A.baum*	Colistin	Amikacin<br>Cefepime<br>Ceftazidime<br>Ciprofloxacin<br>Gentamycin<br>Impenem<br>Meropenem<br>Piperacillin-Tazobactam<br>Tobramycin<br>Trimethoprim-sulfamethoxazole	*A.baum*<br><br>*S.aur*	Colistin<br><br>Clindamycin<br>Fusidic acid<br>Linezolid<br>Rifampicin<br>Vancomycin	Amikacin<br>Cefepime<br>Ceftazidime<br>Ciprofloxacin<br>Gentamycin<br>Impenem<br>Meropenem<br>Piperacillin-Tazobactam<br>Tobramycin<br>Trimethoprim-sulfamethoxazole<br>Chloramphenicol<br>Ciprofloxacin<br>Cloxacillin<br>Erythromycin/ Azithromycin<br>Gentamycin<br>Penicillin/Ampicillin<br>Trimethoprim-sulfamethoxazole	*S.aur*	Negative
9	Mar-16	25-30	Nosocomial sepsis, unspecified bacteria	Ampicillin<br>Gentamycin<br>Tazobactam<br>Amikacin<br>Meropenem<br>Vancomycin<br>Colistin	Negative			Negative			*S.marc*	Amikacin<br>Cefepime<br>Cefotaxime/<br>Ceftriaxone<br>Ceftazidime<br>Chloramphenicol<br>Ciprofloxacin<br>Ertapenem<br>Gentamycin<br>Imipenem<br>Tobramycin<br>1Piperacillin-<br>Tazobactam<br>1Trimethoprim-<br>sulfamethoxazole	Amoxicillin-clavulanic acid<br>Ampicillin/Amoxicillin<br>Cefazolin<br>Cefuroxime	Negative	Negative
10	Jun-16	5-10	Gram negative, nosocomial sepsis	Ampicillin<br>Gentamycin	Negative			*A.baum*	Colistin	Amikacin<br>Cefepime<br>Ceftazidime<br>Ciprofloxacin<br>Gentamycin<br>Impenem<br>Meropenem<br>Piperacillin-Tazobactam<br>Tobramycin<br>Trimethoprim-sulfamethoxazole	*A.baum*	Colistin	Amikacin<br>Cefepime<br>Ceftazidime<br>Ciprofloxacin<br>Gentamycin<br>Impenem<br>Meropenem<br>Piperacillin-Tazobactam<br>Tobramycin<br>Trimethoprim-sulfamethoxazole	Negative	Negative
11	Jun-16	1-5	RDS of newborn	Ampicillin<br>Gentamycin	Negative			Negative			*A.baum*	Colistin	Amikacin<br>Cefepime<br>Ceftazidime<br>Ciprofloxacin<br>Gentamycin<br>Impenem<br>Meropenem<br>Piperacillin-Tazobactam	Negative	Negative

													Tobramycin<br>Trimethoprim-sulfamethoxazole		
12	Jul-16	5-10	Nosocomial bacterial meningitis	Ampicillin<br>Gentamycin<br>Tazobactam<br>Amikacin<br>Meropenem	*A.baum*	Colistin		*A.baum*	Colistin	Amikacin<br>Cefepime<br>Ceftazidime<br>Ciprofloxacin<br>Gentamycin<br>Impenem<br>Meropenem<br>Piperacillin-Tazobactam<br>Tobramycin<br>Trimethoprim-sulfamethoxazole	*A.baum*	Colistin	Amikacin<br>Cefepime<br>Ceftazidime<br>Ciprofloxacin<br>Gentamycin<br>Impenem<br>Meropenem<br>Piperacillin-Tazobactam<br>Tobramycin<br>Trimethoprim-sulfamethoxazole	Negative	Negative
13	Feb-16	1-5	Gram negative nosocomial Sepsis	Ampicillin<br>Gentamycin	*A.baum*	Colistin	Amikacin<br>Cefepime<br>Ceftazidime<br>Ciprofloxacin<br>Gentamycin<br>Impenem<br>Meropenem<br>Piperacillin-Tazobactam<br>Tobramycin<br>Trimethoprim-sulfamethoxazole	*A.baum*	Colistin	Amikacin<br>Cefepime<br>Ceftazidime<br>Ciprofloxacin<br>Gentamycin<br>Impenem<br>Meropenem<br>Piperacillin-Tazobactam<br>Tobramycin<br>Trimethoprim-sulfamethoxazole	*A.baum*	Colistin	Amikacin<br>Cefepime<br>Ceftazidime<br>Ciprofloxacin<br>Gentamycin<br>Impenem<br>Meropenem<br>Piperacillin-Tazobactam<br>Tobramycin<br>Trimethoprim-sulfamethoxazole	Negative	Negative
14	Jun-16	5-10	Congenital diaphragmatic hernia	Ampicillin<br>Gentamycin	*A.baum*	Colistin	Amikacin<br>Cefepime<br>Ceftazidime<br>Ciprofloxacin<br>Gentamycin<br>Impenem<br>Meropenem<br>Piperacillin-Tazobactam<br>Tobramycin	Negative			Negative			Negative	*Ureaplasma*
15	Jun-16	5-10	Gram negative nosocomial sepsis	Missing data	Missing data	Missing data	Missing data	*A.baum*	Colistin<br>Tobramycin	Amikacin<br>Cefepime<br>Ceftazidime<br>Ciprofloxacin<br>Gentamycin<br>Impenem<br>Meropenem<br>Piperacillin-Tazobactam<br>Trimethoprim-sulfamethoxazole	Negative			*K.pneu*	Negative
16	Feb-16	5-10	Gram negative nosocomial sepsis	Ampicillin<br>Gentamycin	*S.aur*<br><br><br><br><br><br><br>*A.baum*	Clindamycin<br>Fusidic acid<br>Linezolid<br>Rifampicin<br>Vancomycin<br><br><br>Colistin	Chloramphenicol<br>Ciprofloxacin<br>Cloxacillin<br>Erythromycin/ Azithromycin<br>Gentamycin<br>Penicillin/Ampicillin<br>Trimethoprim-sulfamethoxazole<br>Amikacin<br>Cefepime<br>Ceftazidime<br>Ciprofloxacin<br>Gentamycin<br>Impenem<br>Meropenem<br>Piperacillin-Tazobactam<br>Tobramycin<br>Trimethoprim-sulfamethoxazole	Negative			*S.aur*<br><br><br><br><br><br><br>*A.baum*	Clindamycin<br>Fusidic acid<br>Linezolid<br>Rifampicin<br>Vancomycin<br><br><br>Colistin	Chloramphenicol<br>Ciprofloxacin<br>Cloxacillin<br>Erythromycin/ Azithromycin<br>Gentamycin<br>Penicillin/Ampicillin<br>Trimethoprim-sulfamethoxazole<br>Amikacin<br>Cefepime<br>Ceftazidime<br>Ciprofloxacin<br>Gentamycin<br>Impenem<br>Meropenem<br>Piperacillin-Tazobactam<br>Tobramycin<br>Trimethoprim-sulfamethoxazole	*S.aur*	*S.aur*
17	Jul-15	5-10	Gram negative nosocomial Sepsis	Ampicillin<br>Gentamycin	Negative			Negative			*A.baum*	Colistin	Amikacin<br>Cefepime<br>Ceftazidime<br>Ciprofloxacin<br>Gentamycin<br>Impenem<br>Meropenem<br>Piperacillin-Tazobactam<br>Tobramycin	Negative	Negative

													Trimethoprim-sulfamethoxazole		
18	Dec-15	15-20	Nosocomial K.pneu Sepsis	Ampicillin<br>Gentamycin<br>Tazobactam<br>Amikacin	*K.pneu*	Ertapenem<br>Imipenem	Ampicillin/Amoxicillin<br>Amoxicillin-clavulanic acid<br>Ciprofloxacin<br>Cefazolin<br>Cefuroxime (Parenteral)<br>Cefotaxime/Ceftriaxone<br>Ceftazidime<br>Cefepime<br>Gentamicin<br>Piperacillin-tazobactam<br>Tobramycin<br>Trimethoprim-sulfamethoxazole	Negative			*K.pneu*<br>CNS	Ertapenem<br>Imipenem	Ampicillin/Amoxicillin<br>Amoxicillin-clavulanic acid<br>Ciprofloxacin<br>Cefazolin<br>Cefuroxime (Parenteral)<br>Cefotaxime/Ceftriaxone<br>Ceftazidime<br>Cefepime<br>Gentamicin<br>Piperacillin-tazobactam<br>Tobramycin<br>Trimethoprim-sulfamethoxazole	Negative	CMV, *S.aur*
19	Feb-16	5-10	Nosocomial MRSA sepsis	Ampicillin<br>Gentamycin	*S.aur*	Clindamycin<br>Fusidic acid<br>Linezoid<br>Rifampicin<br>Vancomycin	Chloramphenicol<br>Cloxacillin<br>Erythromycin/ Azithromycin<br>Gentamycin<br>Penicillin/Ampicillin<br>Trimethoprim-sulfamethoxazole	MRSA	Clindamycin<br>Fusidic acid<br>Linezoid<br>Rifampicin<br>Vancomycin	Chloramphenicol<br>Cloxacillin<br>Erythromycin/ Azithromycin<br>Gentamycin<br>Penicillin/Ampicillin<br>Trimethoprim-sulfamethoxazole	MRSA	Clindamycin<br>Fusidic acid<br>Linezoid<br>Rifampicin<br>Vancomycin	Chloramphenicol<br>Cloxacillin<br>Erythromycin/ Azithromycin<br>Gentamycin<br>Penicillin/Ampicillin<br>Trimethoprim-sulfamethoxazole	*S.aur*	S.aur
20	Jan-16	10-15	Nosocomial gram negative sepsis	Ampicillin<br>Gentamycin<br>Vancomycin	*K.pneu, C.albicans*	Amikacin<br>Chloramphenicol<br>Ertapenem<br>Imipenem<br>Meropenem<br>Nalidix acid<br>Piperacillin-Tazobactam	Amoxicillin-clavulanic acid<br>Ampicillin/Amoxicillin<br>Cefazolin<br>Cefepime<br>Cefotaxime/ Ceftriaxone<br>Cefoxitin<br>Ceftazidime<br>Cefuroxime<br>Ciprofloxacin<br>Gentamycin<br>Tobramycin<br>Trimethoprim-sulfamethoxazole	*K.pneu*	Amikacin<br>Chloramphenicol<br>Ertapenem<br>Imipenem<br>Meropenem<br>Nalidix acid<br>Piperacillin-Tazobactam	Amoxicillin-clavulanic acid<br>Ampicillin/Amoxicillin<br>Cefazolin<br>Cefepime<br>Cefotaxime/ Ceftriaxone<br>Cefoxitin<br>Ceftazidime<br>Cefuroxime<br>Ciprofloxacin<br>Gentamycin<br>Tobramycin<br>Trimethoprim-sulfamethoxazole	Negative			*K.pneu*	Negative
21	Nov-15	15-20	Nosocomial gram negative sepsis	Ampicillin<br>Gentamycin<br>Tazobactam<br>Amikacin	Negative			*K.pneu*	Amikacin<br>Cefoxitin<br>Ciprofloxacin<br>Ertapenem<br>Imipenem<br>Meropenem<br>Nalidix acid<br>Piperacillin-Tazobactam<br>Tobramycin	Amoxicillin-clavulanic acid<br>Ampicillin/Amoxicillin<br>Cefazolin<br>Cefepime<br>Ceftazidime<br>Cefuroxime<br>Gentamycin<br>Trimethoprim-sulfamethoxazole	Negative			*K.pneu*	*S.aur, E.coli*
22	Nov-15	1-5	RDS of newborn	Ampicillin<br>Gentamycin	Negative			Negative			Negative			Negative	Negative
23	Nov-15	5-10	Bacterial sepsis of newborn	Ampicillin<br>Gentamycin	Negative			Negative			Negative			Negative	*Clamydia trachomatis*
24	Jan-16	5-10	Bacterial sepsis of newborn	Ampicillin<br>Gentamycin	Negative			Negative			Negative			*H.inf*	Negative
	Jun-16	1-5	Congenital pneumonia, unspecified bacteria	Ampicillin<br>Gentamycin	*Corynebacterium****			*K.oxy*	Amikacin<br>Amoxicillin-clavulanic acid<br>Cefazolin<br>Cefepime<br>Cefotaxime/ Ceftriaxone<br>Cefoxitin<br>Ceftazidime<br>Cefuroxime<br>Chloramphenicol<br>Ciprofloxacin<br>Ertapenem<br>Imipenem<br>Tobramycin	Ampicillin/Amoxicillin	*K.oxy*<br>*A.baum*	Amikacin<br>Amoxicillin-clavulanic acid<br>Cefazolin<br>Cefepime<br>Cefotaxime/ Ceftriaxone<br>Cefoxitin<br>Ceftazidime<br>Cefuroxime<br>Chloramphenicol<br>Ciprofloxacin<br>Ertapenem<br>Imipenem<br>Tobramycin<br>Colistin	Ampicillin/Amoxicillin<br>Amikacin	Negative	Negative

													Cefepime<br>Ceftazidime<br>Ciprofloxacin<br>Gentamycin<br>Impenem<br>Meropenem<br>Piperacillin-Tazobactam<br>Tobramycin<br>Trimethoprim-sulfamethoxazole		
26	Jul-16	1-5	Bacterial sepsis of newborn, unspecified bacteria	Ampicillin<br>Gentamycin<br>Tazobactam<br>Amikacin	Negative			*A.baum*	Colistin<br>Tobramycin	Amikacin<br>Cefepime<br>Ceftazidime<br>Ciprofloxacin<br>Gentamycin<br>Impenem<br>Meropenem<br>Piperacillin-Tazobactam<br>Trimethoprim-sulfamethoxazole	Negative			Negative	Negative
27	Apr-16	1-5	Nosocomial sepsis, unspecified bacteria	Ampicillin<br>Gentamycin	Negative			Negative			CNS			Negative	Negative
28	Apr-16	5-10	Nosocomial sepsis, unspecified bacteria	Ampicillin<br>Gentamycin	Negative			Negative			Negative			*S.aur*	Negative
29	Apr-16	5-10	Nosocomial sepsis	Ampicillin<br>Gentamycin<br>Tazobactam<br>Amikacin	Negative			Negative			MRSA	Clindamycin<br>Fusidic acid<br>Linezoid<br>Rifampicin<br>Vancomycin	Chloramphenicol<br>Cloxacillin<br>Erythromycin/ Azithromycin<br>Gentamycin<br>Penicillin/Ampicillin<br>Trimethoprim-sulfamethoxazole	*S.aur*	*S.aur*
30	Jun-16	5-10	Nosocomial sepsis, unspecified bacteria	Ampicillin<br>Gentamycin	Negative			Negative			Negative			Negative	Negative
31	Jul-17	1-5	Nosocomial sepsis, unspecified bacteria	Ampicillin<br>Gentamycin<br>Meropenem<br>Vancomycin	Negative			*P.mira*			Negative			Negative	Negative

*As determined by the Determination of Cause of Death (DeCoDe) panel of experts reviewed all available medical and laboratory reports. ** indicate where whole genomes were generated. Abbreviations: LRTI: Lower respiratory tract infection, RDS: respiratory distress syndrome; A.baum: *Acinetobacter baumannii*, K.pneu: *Klebsiella pneumonia*, E.coli: *Escherichia coli*, RSV: respiratory syncytial virus, CMV: cytomegalovirus, S.aur: *Staphylococcus aureus*, K.aero: *Klebsiella aerogenes*, S.Marc: *Serratia marcescens*, S.haem: *Staphylococcus haemolyticus,* MRSA: Methicillin-resistant *Staphylococcus aureus, haemolyticus,* H.haem*: Haemophilus haemolyticus;* S.mitis: *Streptococcus mitis;* P.mira: *Proteus mirabilis; U.urealyticum: Ureaplasma urealyticum*
