# Supplementary material for: Metagenomic sequencing of post-mortem tissue samples for the identification of pathogens associated with neonatal deaths
Source: Nat Commun. 2023 Sep 4;14:5373. doi: 10.1038/s41467-023-40958-8 (PMC10477270; doi:10.1038/s41467-023-40958-8)
Supplement: Supplementary file 3 — Reporting Summary [file 41467_2023_40958_MOESM3_ESM.pdf]

nature portfolio

Corresponding author(s): Vicky L Baillie

Last updated by author(s): May 29, 2023

Reporting Summary

Nature Portfolio wishes to improve the reproducibility of the work that we publish. This form provides structure for consistency and transparency in reporting. For further information on Nature Portfolio policies, see our Editorial Policies and the Editorial Policy Checklist.

Statistics

For all statistical analyses, confirm that the following items are present in the figure legend, table legend, main text, or Methods section.

n/a	Confirmed	
☐	☒	The exact sample size (*n*) for each experimental group/condition, given as a discrete number and unit of measurement
☐	☒	A statement on whether measurements were taken from distinct samples or whether the same sample was measured repeatedly
☐	☒	The statistical test(s) used AND whether they are one- or two-sided<br>*Only common tests should be described solely by name; describe more complex techniques in the Methods section.*
☒	☐	A description of all covariates tested
☒	☐	A description of any assumptions or corrections, such as tests of normality and adjustment for multiple comparisons
☐	☒	A full description of the statistical parameters including central tendency (e.g. means) or other basic estimates (e.g. regression coefficient) AND variation (e.g. standard deviation) or associated estimates of uncertainty (e.g. confidence intervals)
☐	☒	For null hypothesis testing, the test statistic (e.g. *F*, *t*, *r*) with confidence intervals, effect sizes, degrees of freedom and *P* value noted<br>*Give P values as exact values whenever suitable.*
☒	☐	For Bayesian analysis, information on the choice of priors and Markov chain Monte Carlo settings
☒	☐	For hierarchical and complex designs, identification of the appropriate level for tests and full reporting of outcomes
☒	☐	Estimates of effect sizes (e.g. Cohen's *d*, Pearson's *r*), indicating how they were calculated

Our web collection on statistics for biologists contains articles on many of the points above.

Software and code

Policy information about availability of computer code

Data collection	REDCap electronic data capture tools hosted at the University of the Witwatersrand.
Data analysis	STATA Version 11.0 (StataCorp, Texas, USA)

For manuscripts utilizing custom algorithms or software that are central to the research but not yet described in published literature, software must be made available to editors and reviewers. We strongly encourage code deposition in a community repository (e.g. GitHub). See the Nature Portfolio guidelines for submitting code & software for further information.

Data

Policy information about availability of data

All manuscripts must include a data availability statement. This statement should provide the following information, where applicable:

- Accession codes, unique identifiers, or web links for publicly available datasets
- A description of any restrictions on data availability
- For clinical datasets or third party data, please ensure that the statement adheres to our policy

All data needed to evaluate the conclusions of this work are present in the main document and/or the Supplementary Materials.

Research involving human participants, their data, or biological material

Policy information about studies with human participants or human data. See also policy information about sex, gender (identity/presentation), and sexual orientation and race, ethnicity and racism.

Reporting on sex and gender	Information was collected in the parent study but was not used in this manuscript.
Reporting on race, ethnicity, or other socially relevant groupings	None of these variables are reported on in this manuscript.
Population characteristics	None of these variables are reported on in this manuscript.
Recruitment	Any stillbirth, neonate or paediatric death that occurred in the hospital were eligible for enrollment into the study. Only a subset of these were included in the analysis based on decedents that had residual samples available for testing. This was completely random and all samples with sufficient residual samples were analyzed.
Ethics oversight	Human Research Ethics Committee of the University of the Witwatersrand

Note that full information on the approval of the study protocol must also be provided in the manuscript.

Field-specific reporting

Please select the one below that is the best fit for your research. If you are not sure, read the appropriate sections before making your selection.

☒ Life sciences ☐ Behavioural & social sciences ☐ Ecological, evolutionary & environmental sciences

For a reference copy of the document with all sections, see nature.com/documents/nr-reporting-summary-flat.pdf

Life sciences study design

All studies must disclose on these points even when the disclosure is negative.

Sample size	No sample size calculation was performed as we used a convenient sample size of cases and controls were sufficient sample was available for metagenomic next generation sequencing. This was a proof of concept type study - we wanted to determine whether mNGS could be used to determine the cause of death and we believe the sample size was sufficient for this purpose.
Data exclusions	No data was excluded
Replication	We compared the results of the mNGS testing compared to gold standard testing (conventional microbiological culturing) and reported on the agreement between the two methods.
Randomization	This was no relevant to our study. All neonatal decedents were eligible for enrolment into the parent study. A panel of expert then reviewed all data to determine the cause of death. This study then looked at neonates that died of non infectious causes (controls) and infectious causes (cases) based on the expert panels determinations.
Blinding	During all testing and bioinformatic analysis the scientists were blinded to the causes of death.

Reporting for specific materials, systems and methods

We require information from authors about some types of materials, experimental systems and methods used in many studies. Here, indicate whether each material, system or method listed is relevant to your study. If you are not sure if a list item applies to your research, read the appropriate section before selecting a response.

Materials & experimental systems

n/a	Involved in the study	
☒	☐	Antibodies
☒	☐	Eukaryotic cell lines
☒	☐	Palaeontology and archaeology
☒	☐	Animals and other organisms
☒	☐	Clinical data
☒	☐	Dual use research of concern
☒	☐	Plants

Methods

n/a	Involved in the study	
☒	☐	ChIP-seq
☒	☐	Flow cytometry
☒	☐	MRI-based neuroimaging
